# Neoadjuvant osimertinib and chemotherapy for stage IIIA primary pulmonary carcinosarcoma with EGFR 19DEL mutation: A case report

**DOI:** 10.3389/fonc.2023.1145021

**Published:** 2023-03-10

**Authors:** Hongming Wang, Zhijun Wu, Yangfeng Du, Tao Wu, Wei Tian, Wen Dong, Juan Cai, Jiang Zheng, Yan Zhang, Shiyan Li, Wei Xu, Jing Qin, Zemin Xiao

**Affiliations:** ^1^ Department of Oncology, The First People’s Hospital of Changde City, Changde, China; ^2^ Department of Thoracic Surgery, The First People’s Hospital of Changde City, Changde, China; ^3^ Department of Pathology, The First People’s Hospital of Changde City, Changde, China

**Keywords:** osimertinib, chemotherapy, neoadjuvant, pulmonary carcinosarcoma, EGFR

## Abstract

Epidermal growth factor receptor (EGFR) mutations have been frequently detected in patients with pulmonary adenocarcinoma. EGFR Exon 19Del and 21L858R mutations are the two most common EGFR mutations. EGFR-tyrosine kinase inhibitors (TKIs) are widely employed to treat patients with non-small cell lung cancer (NSCLC) harboring EGFR mutations. Recently, there has been rapid growth in clinical trials assessing neoadjuvant targeted therapy, indicating good application prospects owing to high efficiency and low toxicity. Herein, we discuss the case of a 56-year-old male patient who was initially diagnosed with stage IIIA pulmonary adenocarcinoma (AJCC,8^th^ edition) of the left lower lung with an EGFR Exon 19Del mutation. The patient was treated with osimertinib but failed to undergo timely review and surgery. Subsequently, the patient underwent two cycles of neoadjuvant chemotherapy (NAC) combined with neoadjuvant targeted therapy. After the tumor load and size had significantly decreased, radical surgery was successfully performed under thoracoscopy. However, postoperative pathology revealed carcinosarcoma, pT2aN0M0, stage IB, and the pathological response was 50%. The present case report provides practical clinical evidence for the application of neoadjuvant targeted therapy combined with chemotherapy for locally advanced primary pulmonary carcinosarcoma with EGFR mutation.

## 1 Introduction

Osimertinib, the first approved third-generation irreversible selective inhibitor of epidermal growth factor receptor (EGFR) mutations, has been widely used in patients with advanced NSCLC harboring Exon 19Del/21L858R mutations. Osimertinib reportedly exhibits marked efficacy in untreated patients with EGFR mutations, especially those with the EGFR Exon 19Del mutation, thereby affording a longer progression-free survival than first-generation EGFR-TKIs with a similar safety profile (1). On April 14, 2021, the China National Medical Products Administration officially approved the application of osimertinib for the adjuvant treatment of patients with stage IB-IIIA NSCLC harboring EGFR Exon 19Del/21L858R mutations. Considering a prospective clinical trial assessing neoadjuvant targeted therapy, preliminary results have revealed that osimertinib affords substantial clinical effects and good safety, reducing the complexity and scope of surgical resection and improving surgical efficacy (2).

According to the World Health Organization (WHO) classification of thoracic tumors (2021), pulmonary carcinosarcoma (PCS) is a rare type of pulmonary sarcomatoid carcinoma (PSC), accounting for only 4% of PSCs and approximately 0.27% of malignant lung tumors, associated with poor prognosis (3). PCS is more common in middle-aged and elderly male patients than that in female patients, and most patients typically have a prolonged history of heavy smoking. PCS is a special category of lung malignancy with malignant epithelial and mesenchymal components, either clearly demarcated or mixed. Malignant epithelial components mainly include squamous cell carcinoma and adenocarcinoma, whereas malignant mesenchymal components primarily include rhabdomyosarcoma, chondroid sarcoma, and osteosarcoma. Undifferentiated pleomorphic sarcomas are rare. Clinical manifestations are nonspecific, including cough, bloody sputum, chest pain, low fever, emaciation, fatigue, and other discomforts. Chest computed tomography (CT) scans frequently exhibit large lobulated masses prone to bleeding and necrosis. PCS is likely to be misdiagnosed as simple pulmonary carcinoma or sarcoma upon both bronchoscopic biopsy and peripheral puncture biopsy owing to the limited sample size; thus, pathological examination with complete excision is needed to further confirm the diagnosis.

## 2 Case report

**Figure 1:**
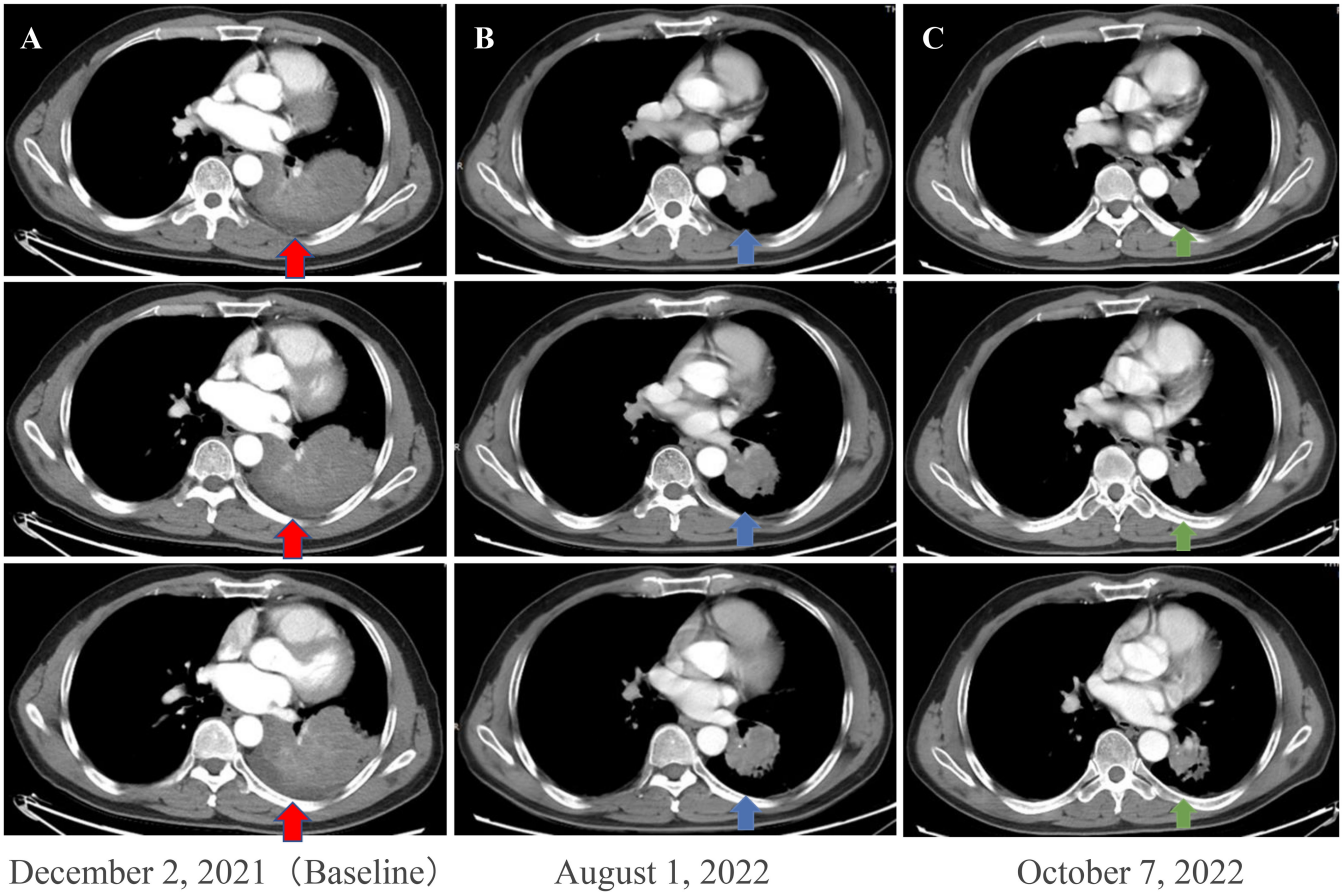
Enhanced chest CT scans of the patient during neoadjuvant therapy. **(A)** Baseline imaging demonstrating a 93 mm × 70 mm abnormal lung mass (red arrows) in the lower lobe of the left lung. **(B)** After 210 days of osimertinib therapy, the chest CT scan shows mass shrinkage (blue arrows) to 48 × 44 mm, achieving a partial response (PR). **(C)** After two cycles (42 days) of osimertinib and chemotherapy, the chest CT scan shows mass shrinkage (green arrows) to 40 mm × 37 mm, achieving a sustained partial response (sPR). CT, computed tomography.

**Figure 2:**
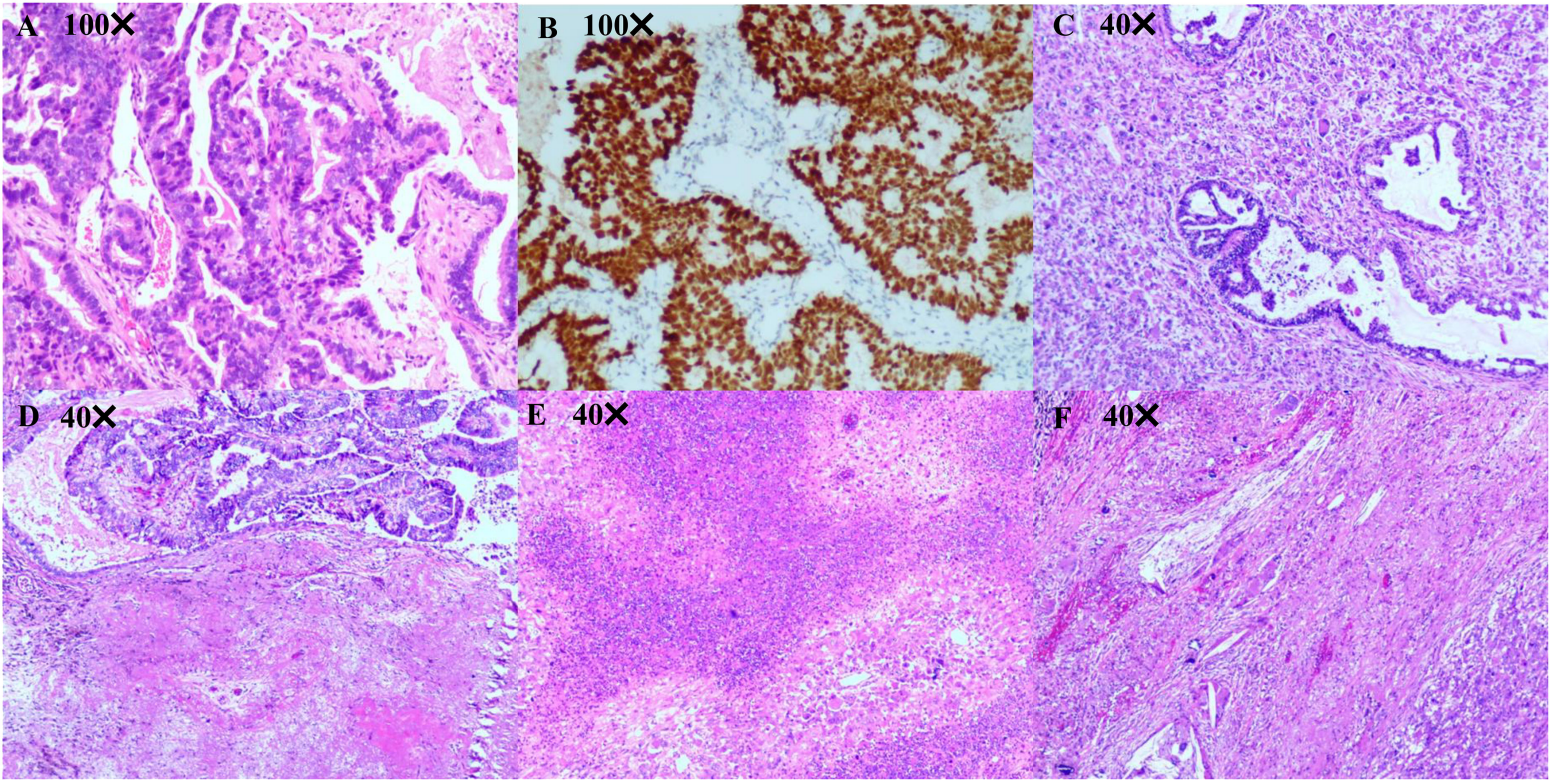
Pathological diagnosis. **(A, B)** Pathological diagnosis of the lung biopsy revealing a moderately differentiated lung adenocarcinoma. Immunohistochemistry results are presented as follows: TTF-1(+), Napsin A (+), CK7 (+), CK5/6 (-), P40 (-), Ki67 (50%+), Syn (-), and CgA (-). **(C-F)** Pathological diagnosis of lung surgery is carcinosarcoma (adenocarcinoma accounts for approximately 40% and undifferentiated pleomorphic sarcoma accounts for approximately 60%) with a size of 40 mm × 33 mm × 26 mm, and the visceral pleura appears uninvolved. No tumor involvement can be observed in the bronchial stump, with no tumor metastasis in the lymph nodes of each group (−, 0/14). Immunohistochemistry outcomes were presented as follows: CK(pan)(partial+), Vimentin(partial+), TTF-1(partial+), CK7 (partial+), NapsinA (partial+), P40 (-), CK5/6 (-), Syn (-), CD56 (few+), S100 (-), SMA (-), Desmin (-), Calponin (-), MyoD1 (-), Myogenin (-), P53 (90%+), Ki67 (60%+), CD31 (partial +), ERG (Vascular +).

A 56-year-old male patient with dry cough, weight loss (2 kg) over 2 months, and a history of smoking (> 35 years), without expectoration, hemoptysis, fever, chills, or dyspnea, was admitted to the Department of Respiratory Medicine at our hospital on December 2, 2021. Family history and physical examination revealed no positive findings. He had an Eastern Cooperative Oncology Group (ECOG) performance status of 1. Enhanced chest CT showed occupation of the left lower lung (approximately 93 mm × 70 mm), with no obvious enlargement of the mediastinal lymph nodes (**Figure 1A**). Electronic bronchoscopy revealed an external pressure stenosis of the basal branch of the lower lobe of the left lung. Ultrasound-guided puncture biopsy of the left lower lung mass revealed moderately differentiated adenocarcinoma (**Figures 2A, B**). No signs of metastasis were detected on upper abdominal enhanced CT, whole-body bone imaging, or brain magnetic resonance imaging (MRI). The tumor was classified as stage IIIA (cT4N0M0). Next-generation sequencing (NGS) analysis (including EGFR, ALK, ROS1, MET, RET, KRAS, BRAF, NRAS, HER2, PIK3CA, and TP53) indicated EGFR Exon 19Del and TP53 mutations (**Supplementary Material**). Following a discussion with the multidisciplinary team at our hospital (including respiratory physicians, oncologists, thoracic surgeons, pathologists, and radiologists), surgical resection combined with adjuvant or neoadjuvant targeted therapy (osimertinib) was recommended. The patient received immediate neoadjuvant targeted therapy (osimertinib 80 mg orally once daily with or without food). However, regular re-examinations and surgery were not performed as required.

**Figure 3:**
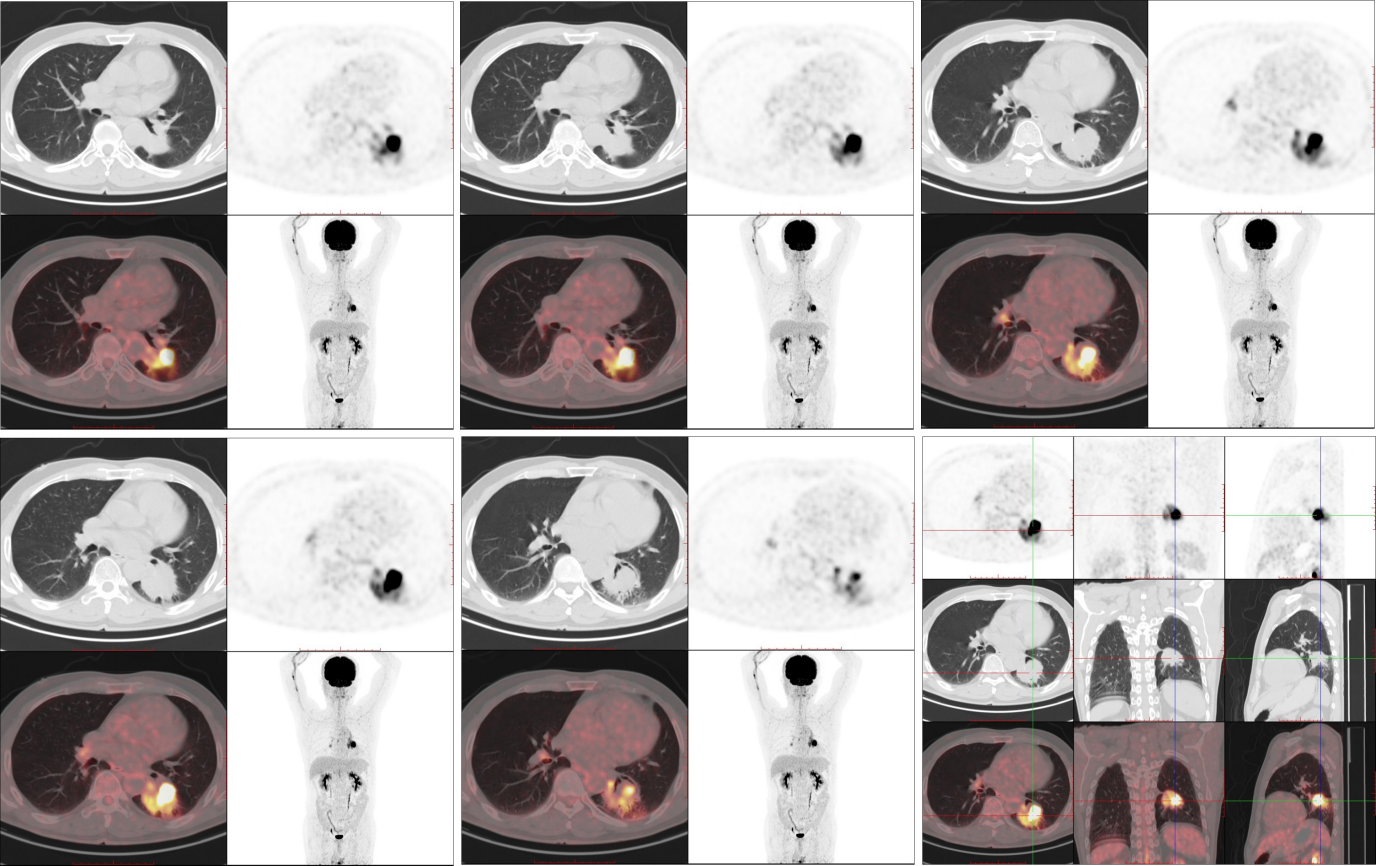
The 18F-FDG PET/CT examination (August 3, 2022). 18-FDG PET/CT shows robust 18-FDG uptake in the left lung mass (SUVmax: 27.998) with a size of 45 × 41 mm; no distant metastasis can be observed.

**Figure 4:**
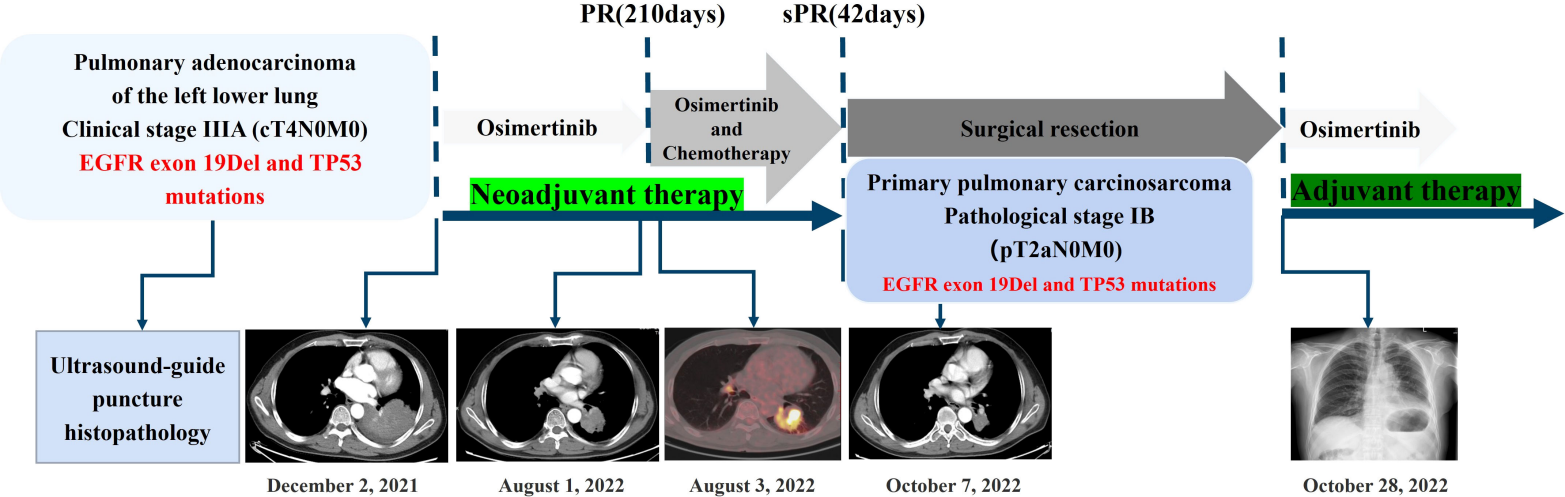
A summary of the treatment strategy employed in this patient.

On August 1, 2022 (210 days after osimertinib therapy), the patient visited the hospital for a re-examination. Based on enhanced chest CT, the tumor in the left lower lung was significantly reduced (**Figure 1B**). Radiographic assessment was partial response (PR) based on the Response Evaluation Criteria in Solid Tumors version 1.1 (RECIST 1.1). Subsequent positron emission tomography (PET)-CT displayed a tumor in the left lower lung accompanied by increased glucose metabolism (SUVmax:27.998) and no other signs of metastasis (**Figure 3**). There was no obvious abnormality on the enhanced brain MRI. Moreover, no notable adverse reactions were observed during osimertinib treatment. Surgery was recommended, and the patient and his family requested consultation before the final determination. From August 25, 2022, to September 16, 2022, the patient received neoadjuvant osimertinib, combined with neoadjuvant chemotherapy, a two-cycle PC regimen (pemetrexed + carboplatin). After chemotherapy, the patient developed moderate gastrointestinal reactions with no obvious myelosuppression. On October 7, 2022, an enhanced chest CT scan showed progressive tumor shrinkage in the left lower lung, exhibiting a size of approximately 40 mm × 37 mm (**Figure 1C**). Radiographic assessment revealed sustained PR (sPR). The urgency of surgery was well-communicated with the patient and his family, and thoracoscopic left lower lobectomy, mediastinal lymph node dissection, and bronchoplasty were successfully completed on October 14, 2022. Postoperative pathological diagnosis was carcinosarcoma with marked necrosis, interstitial degeneration, inflammatory cell infiltration, cholesterol crystals, and small vascular hyperplasia (**Figures 2C-F**). Complete resection was performed, and the pathological response was 50%. The final postoperative pathological stage was pT2aN0M0, stage IB. The patient recovered well post-surgery. Considering the diagnosis of rare primary PCS post-surgery, NGS analysis was re-performed on November 05, 2022, which revealed EGFR Exon 19Del and TP53 mutations (**Supplementary Material**). The patient requested continued adjuvant targeted therapy with osimertinib and regular review. Owing to the impact of the coronavirus disease 2019 (COVID-19) pandemic, the patient was telephonically followed up for three months, exhibiting good health to date (January 14, 2023). The process of clinical diagnosis and treatment for this patient is shown in **Figure 4**.

## 3 Discussion

Although stage IIIA NSCLC is potentially resectable, traditional treatment options, including preoperative or postoperative chemotherapy, offer similar effects (4). Considering patients with resectable NSCLC without known ALK translocations or EGFR mutations, the emergence of neoadjuvant immunotherapy combined with chemotherapy could markedly prolong the event-free survival of patients and improve the pathological complete response (pCR) rate, with no increase in the incidence of adverse events, thereby suggesting survival benefits in patients (5). In the present case report, we recommended neoadjuvant EGFR-TKI treatment for NSCLC owing to the high response rate to osimertinib. However, neoadjuvant targeted therapy for resectable NSCLC with EGFR mutations is currently in its infancy. Based on preliminary studies, EGFR-TKIs have good application prospects in neoadjuvant therapy (2, 6). A phase III, randomized, controlled, multicenter, three-arm study assessing neoadjuvant targeted therapy with EGFR-TKI is ongoing (NeoADAURA) (7). The duration of neoadjuvant targeted therapy was found to vary across different clinical studies and was frequently less than 90 days; the optimal neoadjuvant duration remains uncertain (8, 9).

Considering the present patient, the need for prolonged neoadjuvant targeted therapy could be attributed to poor compliance. However, surgery was not performed despite successful downgrading on the first radiographic assessment of PR after 210 days of osimertinib therapy. In patients with advanced NSCLC harboring EGFR mutations, osimertinib combined with chemotherapy remains safe and tolerable despite increased toxicity (10). Currently, the NeoADAURA study is recruiting patients to evaluate neoadjuvant osimertinib with or without chemotherapy versus chemotherapy alone prior to surgery in patients with operable stage II-IIIB N2 EGFR mutation NSCLC (7). The findings of the NeoADAURA study will likely clarify the most effective combination strategy for neoadjuvant therapy.

TP53 is the most common co-mutant gene in patients with NSCLC carrying EGFR mutations. In addition, TP53/EGFR co-mutations have been associated with poor prognosis (11). Targeted therapy combined with chemotherapy can afford considerable survival benefits in patients with TP53 mutations and a poor prognosis. Moreover, TP53 mutations can shorten the relapse time in postoperative patients who are more likely to benefit from targeted therapy combined with chemotherapy (12). Herein, the postoperative pathology of the patient indicated carcinosarcoma. Establishing whether sarcoma components carry EGFR and TP53 mutations could help further elucidate the pathogenesis of carcinosarcoma and guide postoperative adjuvant therapy. Reportedly, both EGFR and TP53 mutations exhibit a certain mutation frequency in patients with PCS; however, limited patients carry the same gene mutations in both components (13). Related cases have reported that both pulmonary adenocarcinoma and sarcoma components can simultaneously carry EGFR Exon 19Del mutation (14, 15), corroborating the theory of monoclonal histogenesis (13). In the present case report, we successfully separated the carcinoma and sarcoma components using microdissection technology, revealing that both components harbored TP53 and EGFR mutations using NGS (**Supplementary Material**).

In the ADAURA study (16), disease-free survival (DFS) was documented in patients who received adjuvant chemotherapy (hazard ratio [HR] = 0.16, 95% confidence interval [CI]: 0.10–0.26), as well as in those who did not receive adjuvant chemotherapy (HR = 0.23, 95% CI: 0.13–0.40). The authors found that the DFS of the osimertinib group was superior to that of the placebo group regardless of disease stage (stage IB-IIIA). However, the ADAURA study failed to clarify whether the combination adjuvant chemotherapy should be undertaken. The Lung Adjuvant Cisplatin Evaluation (LACE) study (17) revealed that cisplatin-based chemotherapy could significantly improve overall survival (OS) and DFS of the overall population, and the absolute OS rate could be significantly increased by 5.4% in 5 years. However, the OS of stage IB patients was not significantly improved (HR = 0.92, 95% CI: 0.78-1.10). According to the CALGB9633 study (18), some patients with stage IB NSCLC (with high-risk factors) could benefit from postoperative adjuvant chemotherapy. Therefore, adjuvant chemotherapy should not be recommended for stage IB NSCLC except in the presence of pathological risk factors for relapse. In addition, studies have evaluated and demonstrated the potential of circulating tumor DNA-minimal residual disease in predicting the risk of disease recurrence and the benefit of adjuvant chemotherapy post-surgery; however, these results need to be further confirmed in future investigations (19, 20). Moreover, adjuvant immunotherapy may afford limited or uncertain benefits in patients with lung cancer harboring EGFR mutations (21), and related phase II clinical studies are being conducted (22). Large-scale, prospective, phase III, randomized controlled clinical studies are urgently needed for further validation.

In conclusion, this is a successful case of radical surgery after neoadjuvant therapy for stage IIIA PCS with EGFR 19DEL mutation, which also provides specific clinical experience and guidance for perioperative therapy for oncogene-driven NSCLC.

## Data availability statement

The datasets presented in this article are not readily available because of ethical/privacy restrictions. Requests to access the datasets should be directed to the corresponding author.

## Author contributions

HW and ZW collected and analyzed the clinical material and drafted the manuscript. HW, YD, TW, JQ, and WT prepared the figures and **Supplementary Material**. WX performed the surgery. HW, ZW, WD, JC, JZ, SL, and YZ contributed to management and treatment of the patient. ZX revised the final manuscript. All authors contributed to the article and approved the submitted version.

## References

[1] Soria JC Ohe Y Vansteenkiste J Reungwetwattana T Chewaskulyong B Lee KH. Osimertinib in untreated EGFR-mutated advanced non-Small-Cell lung cancer. N Engl J Med (2018) 378(2):113–25. doi: 10.1056/NEJMoa1713137 29151359

[2] Lyu C Fang W Jiao W Ma H Wang J Xu S-D. Osimertinib as neoadjuvant therapy in patients with EGFR mutated resectable stage II-IIIB lung adenocarcinoma (NEOS): Updated results. ELCC (2022) 33:S71–2. doi: 10.1016/j.annonc.2022.02.091 36863124

[3] Sakakura N Uchida T Kitamura Y Suyama M. Pulmonary carcinosarcoma successfully resected using the ribcross thoracotomy approach: report of a case. Surg Today (2014) 44(1):175–9. doi: 10.1007/s00595-012-0357-8 23064966

[4] NSCLC Meta-analysis Collaborative Group. Preoperative chemotherapy for non-small-cell lung cancer: A systematic review and meta-analysis of individual participant data. Lancet (2014) 383(9928):1561–71. doi: 10.1016/S0140-6736(13)62159-5 PMC4022989 24576776

[5] Forde PM Spicer J Lu S Provencio M Mitsudomi T Awad MM. Neoadjuvant nivolumab plus chemotherapy in resectable lung cancer. N Engl J Med (2022) 386(21):1973–85. doi: 10.1056/NEJMoa2202170 PMC9844511 35403841

[6] Zhong WZ Chen KN Chen C Gu CD Wang J Yang XN. Erlotinib versus gemcitabine plus cisplatin as neoadjuvant treatment of stage IIIA-N2 EGFR-mutant non-Small-Cell lung cancer (EMERGING-CTONG 1103): A randomized phase II study. J Clin Oncol (2019) 37(25):2235–45. doi: 10.1200/JCO.19.00075 31194613

[7] Tsuboi M Weder W Escriu C Blakely C He J Dacic S. Neoadjuvant osimertinib with/without chemotherapy versus chemotherapy alone for EGFR-mutated resectable non-small-cell lung cancer: NeoADAURA. Future Oncol (2021) 17(31):4045–55. doi: 10.2217/fon-2021-0549 PMC8530153 34278827

[8] Liu W Ren S Xiao Y Yang L Zeng C Hu Y. Neoadjuvant targeted therapy for resectable EGFR-mutant non-small cell lung cancer: Current status and future considerations. Front Pharmacol (2022) 13:1036334. doi: 10.3389/fphar.2022.1036334 36467102 PMC9712740

[9] Liu SY Zhang JT Zeng KH Wu YL. Perioperative targeted therapy for oncogene-driven NSCLC. Lung Cancer (2022) 172:160–9. doi: 10.1016/j.lungcan.2022.05.007 35644704

[10] Planchard D Feng PH Karaseva N Kim SW Kim TM Lee CK. Osimertinib plus platinum-pemetrexed in newly diagnosed epidermal growth factor receptor mutation-positive advanced/metastatic non-small-cell lung cancer: Safety run-in results from the FLAURA2 study. ESMO Open (2021) 6(5):100271. doi: 10.1016/j.esmoop.2021.100271 34543864 PMC8453202

[11] Wang F Zhao N Gao G Deng HB Wang ZH Deng LL. Prognostic value of TP53 co-mutation status combined with EGFR mutation in patients with lung adenocarcinoma. J Cancer Res Clin Oncol (2020) 146(11):2851–9. doi: 10.1007/s00432-020-03340-5 PMC11804661 32743759

[12] Fu J Tong Y Xu Z Li Y Zhao Y Wang T. Impact of TP53 mutations on EGFR-tyrosine kinase inhibitor efficacy and potential treatment strategy. Clin Lung Cancer (2023) 24(1):29–39. doi: 10.1016/j.cllc.2022.08.007 36117108

[13] Chang YL Wu CT Shih JY Lee YC. EGFR and p53 status of pulmonary pleomorphic carcinoma: Implications for EGFR tyrosine kinase inhibitors therapy of an aggressive lung malignancy. Ann Surg Oncol (2011) 18(10):2952–60. doi: 10.1245/s10434-011-1621-7 21409490

[14] Toyokawa G Takenoyama M Taguchi K Arakaki K Inamasu E Toyozawa R. The first case of lung carcinosarcoma harboring in-frame deletions at exon19 in the EGFR gene. Lung Cancer (2013) 81(3):491–4. doi: 10.1016/j.lungcan.2013.06.013 23891513

[15] Koba H Kimura H Nishikawa S Sone T Abo M Hara J. Next-generation sequencing analysis identifies genomic alterations in pathological morphologies: A case of pulmonary carcinosarcoma harboring EGFR mutations. Lung Cancer (2018) 122:146–50. doi: 10.1016/j.lungcan.2018.05.026 30032823

[16] Wu YL John T Grohe C Majem M Goldman JW Kim SW. Postoperative chemotherapy use and outcomes from ADAURA: Osimertinib as adjuvant therapy for resected EGFR-mutated NSCLC. J Thorac Oncol (2022) 17(3):423–33. doi: 10.1016/j.jtho.2021.10.014 34740861

[17] Pignon JP Tribodet H Scagliotti GV Douillard JY Shepherd FA Stephens RJ. Lung adjuvant cisplatin evaluation: a pooled analysis by the LACE collaborative group. J Clin Oncol (2008) 26(21):3552–9. doi: 10.1200/JCO.2007.13.9030 18506026

[18] Strauss GM. Management of early-stage lung cancer: past, present, and future adjuvant trials. Oncol (Williston Park) (2006) 20(13):1651–63; discussion 1663-4, 1666, 1669-70 passim. 17175743

[19] Qiu B Guo W Zhang F Lv F Ji Y Peng Y. Dynamic recurrence risk and adjuvant chemotherapy benefit prediction by ctDNA in resected NSCLC. Nat Commun (2021) 12(1):6770. doi: 10.1038/s41467-021-27022-z 34799585 PMC8605017

[20] Chaudhuri AA Chabon JJ Lovejoy AF Newman AM Stehr H Azad TD. Early detection of molecular residual disease in localized lung cancer by circulating tumor DNA profiling. Cancer Discov (2017) 7(12):1394–403. doi: 10.1158/2159-8290.CD-17-0716 PMC5895851 28899864

[21] Saw SP Ang MK Tan DS. Adjuvant immunotherapy in patients with early-stage non-small cell lung cancer and future directions. Curr Treat Options Oncol (2022) 23(12):1721–31. doi: 10.1007/s11864-022-01034-3 36451063

[22] Shibaki R Akamatsu H Kato T Nishino K Okada M Mitsudomi T. A phase II study of cisplatin plus vinorelbine combined with atezolizumab as adjuvant therapy for completely resected non-small-cell lung cancer with EGFR mutation (West Japan oncology group 11719L/ADJUST study). Ther Adv Med Oncol (2021) 13:1758835920987647. doi: 10.1177/1758835920987647 33613698 PMC7841658

