# Supplementary material for: Neoadjuvant osimertinib and chemotherapy for stage IIIA primary pulmonary carcinosarcoma with EGFR 19DEL mutation: A case report
Source: Front Oncol. 2023 Mar 10;13:1145021. doi: 10.3389/fonc.2023.1145021 (PMC10036817; doi:10.3389/fonc.2023.1145021)
Supplement: Supplementary file 1 [file Presentation_1.pptx]

## Slide 1
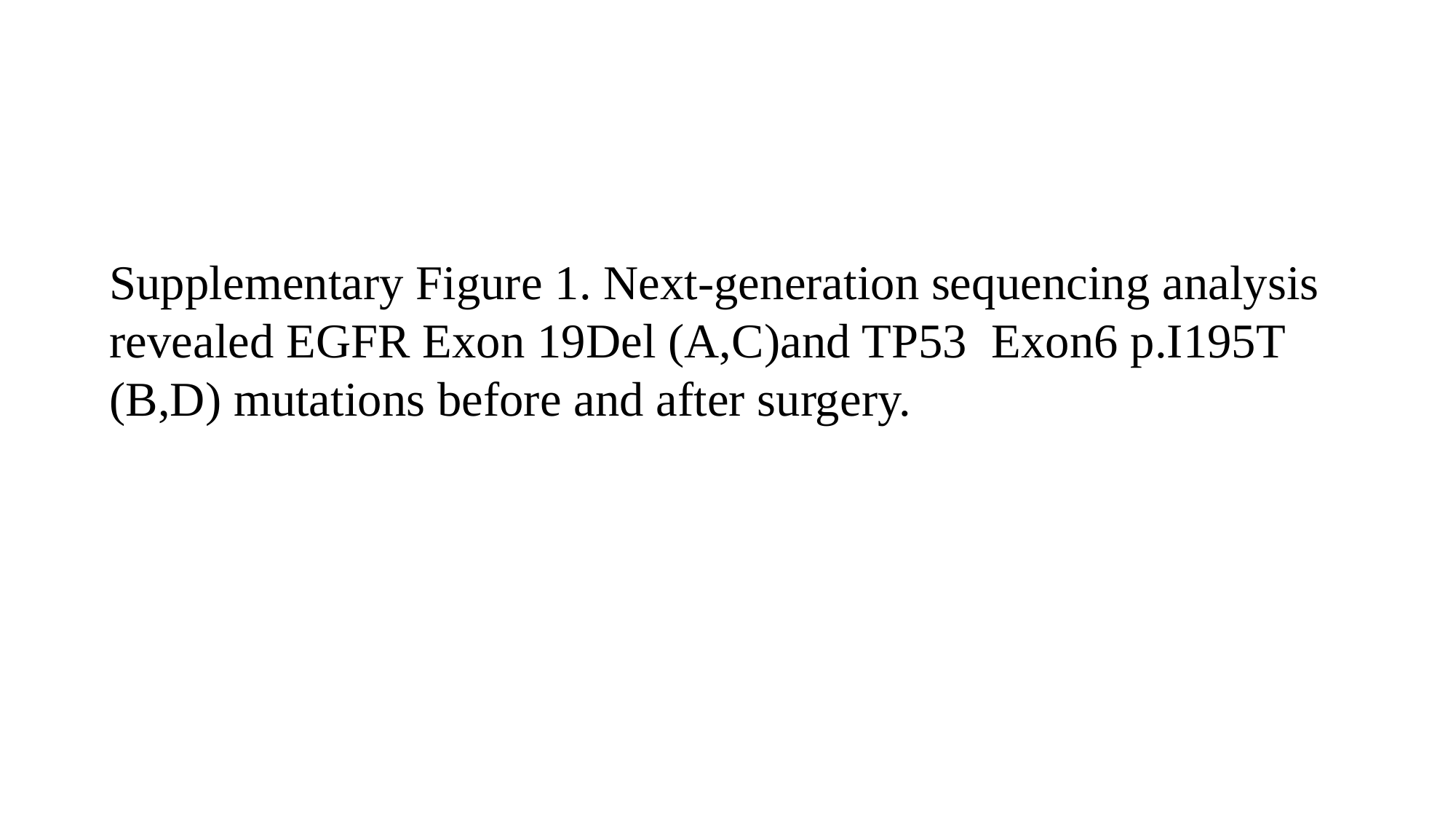

Supplementary Figure 1. Next-generation sequencing analysis revealed EGFR Exon 19Del (A,C)and TP53 Exon6 p.I195T (B,D) mutations before and after surgery.

## Slide 2
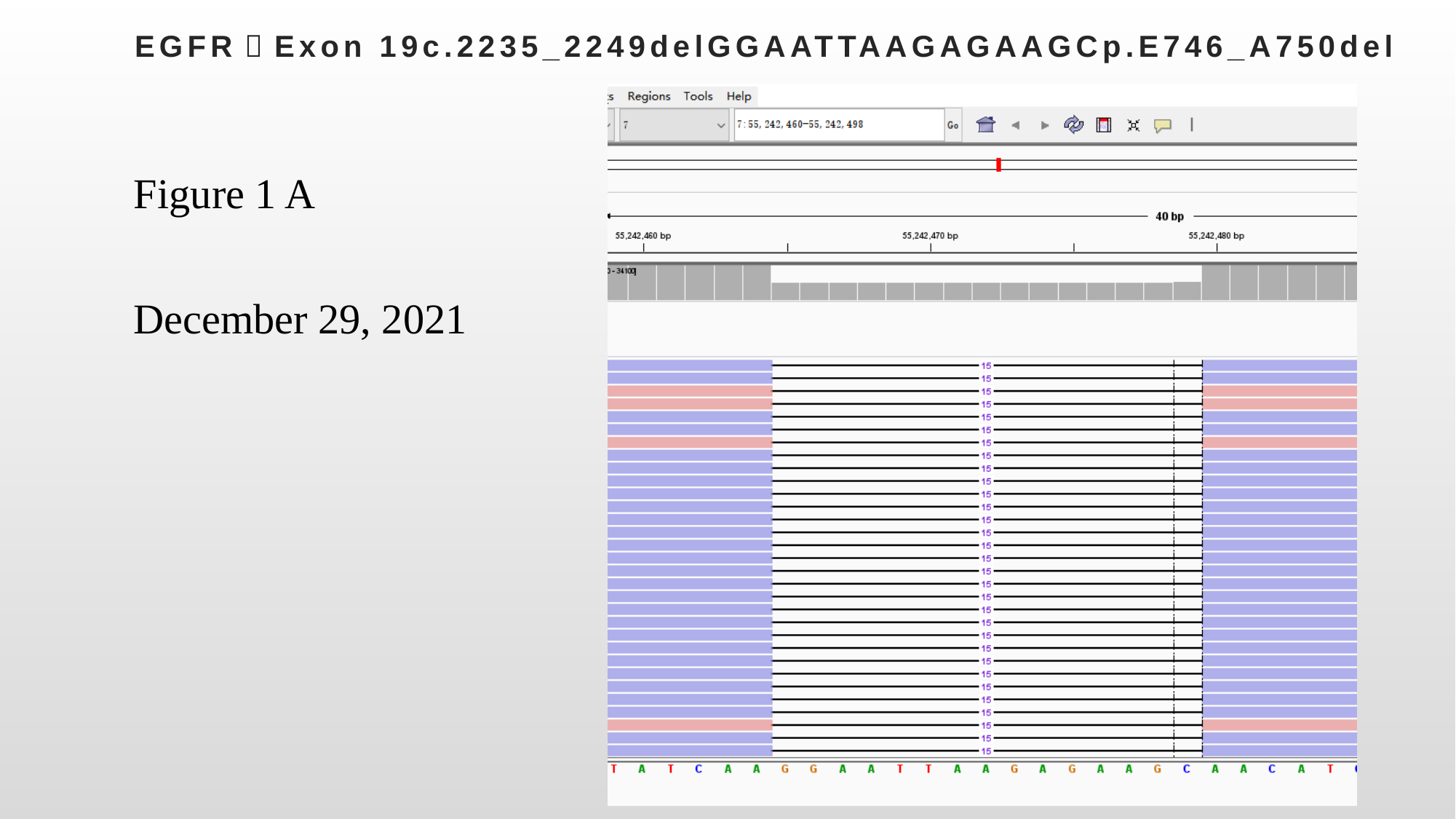

# EGFR：Exon 19c.2235_2249delGGAATTAAGAGAAGCp.E746_A750del
Figure 1 A
December 29, 2021

## Slide 3
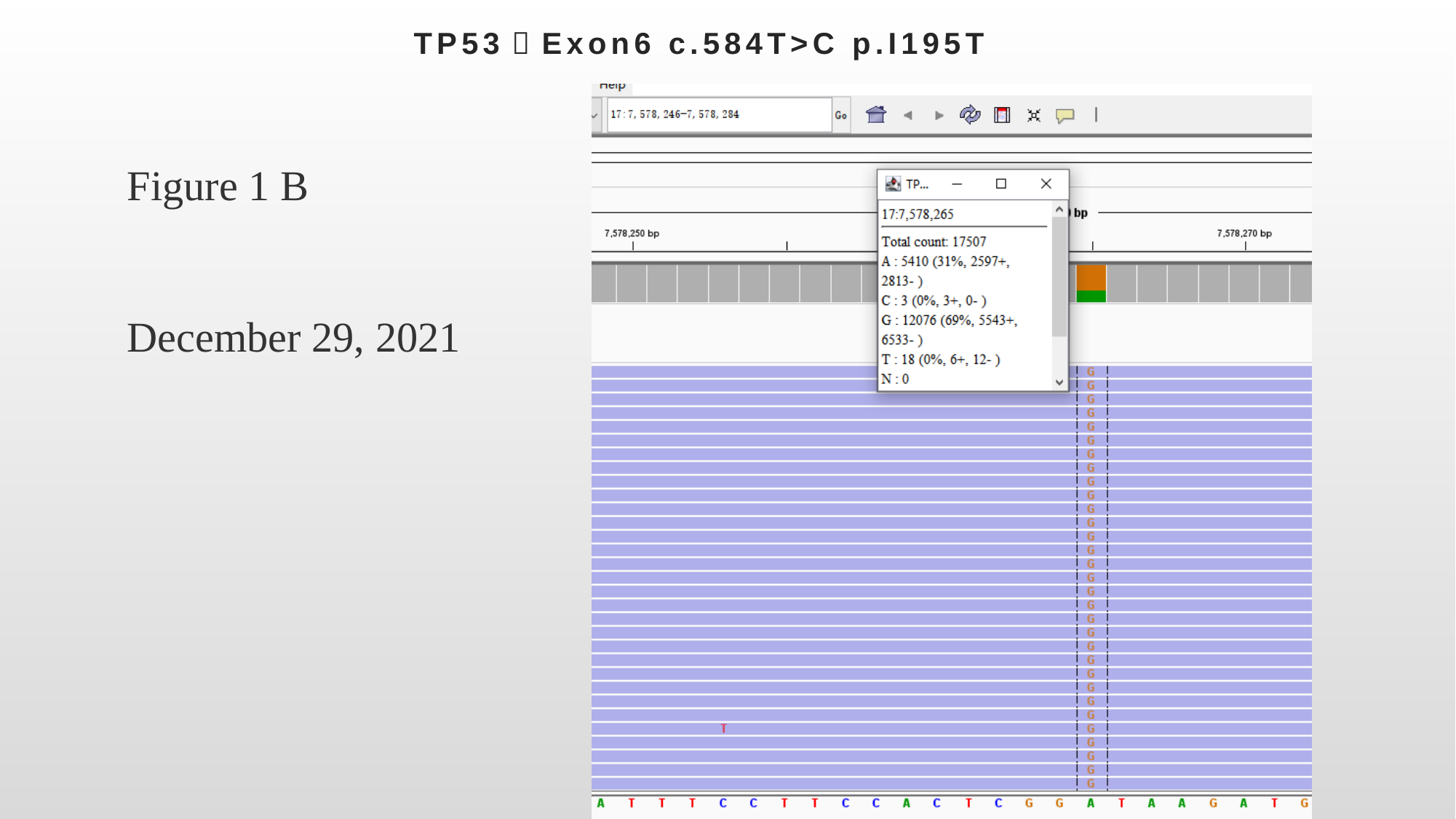

# TP53：Exon6 c.584T>C p.I195T
Figure 1 B
December 29, 2021

## Slide 4
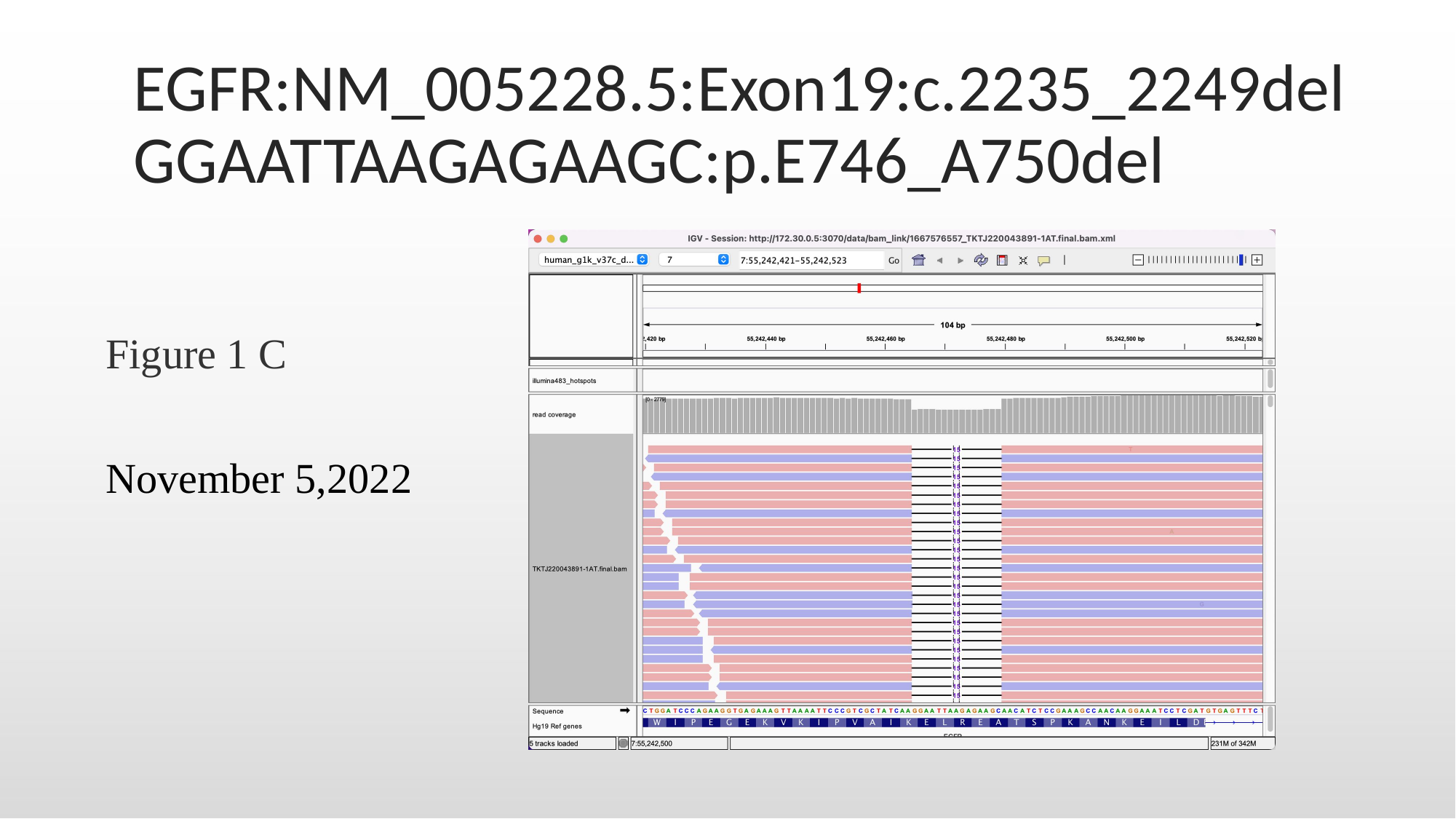

# EGFR:NM_005228.5:Exon19:c.2235_2249delGGAATTAAGAGAAGC:p.E746_A750del
Figure 1 C
November 5,2022

## Slide 5
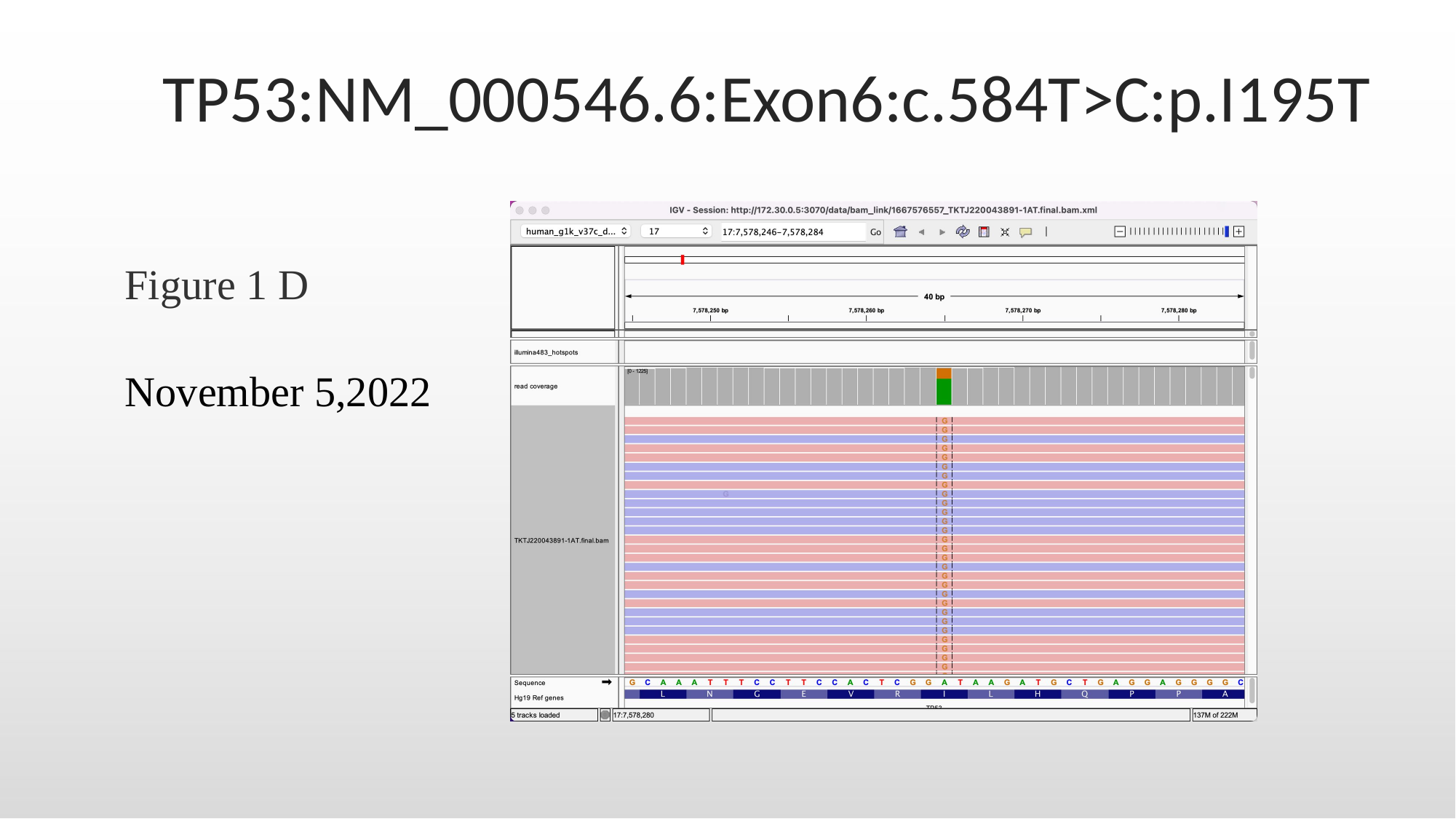

# TP53:NM_000546.6:Exon6:c.584T>C:p.I195T
Figure 1 D
November 5,2022

## Slide 6
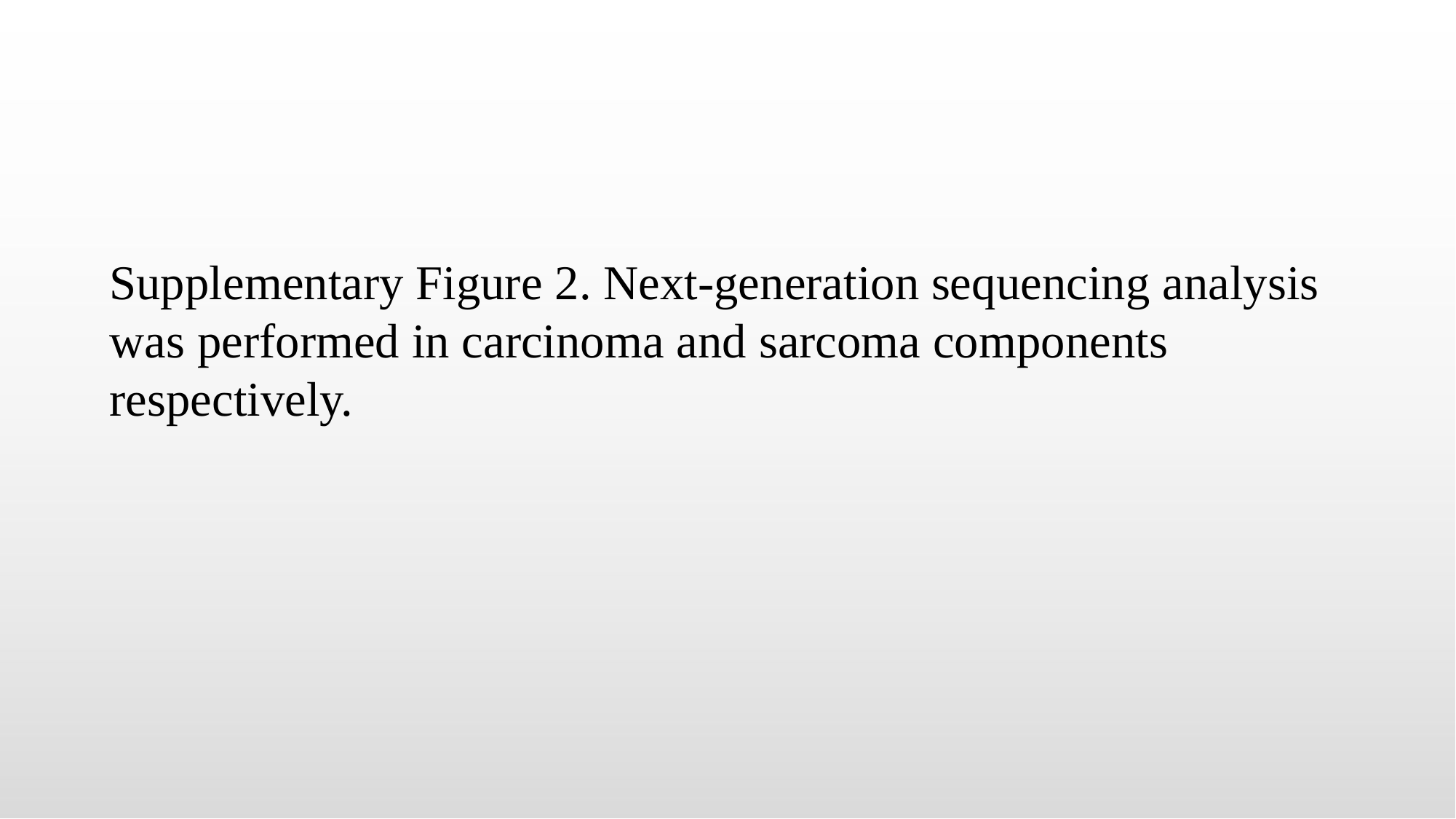

Supplementary Figure 2. Next-generation sequencing analysis was performed in carcinoma and sarcoma components respectively.

## Slide 7
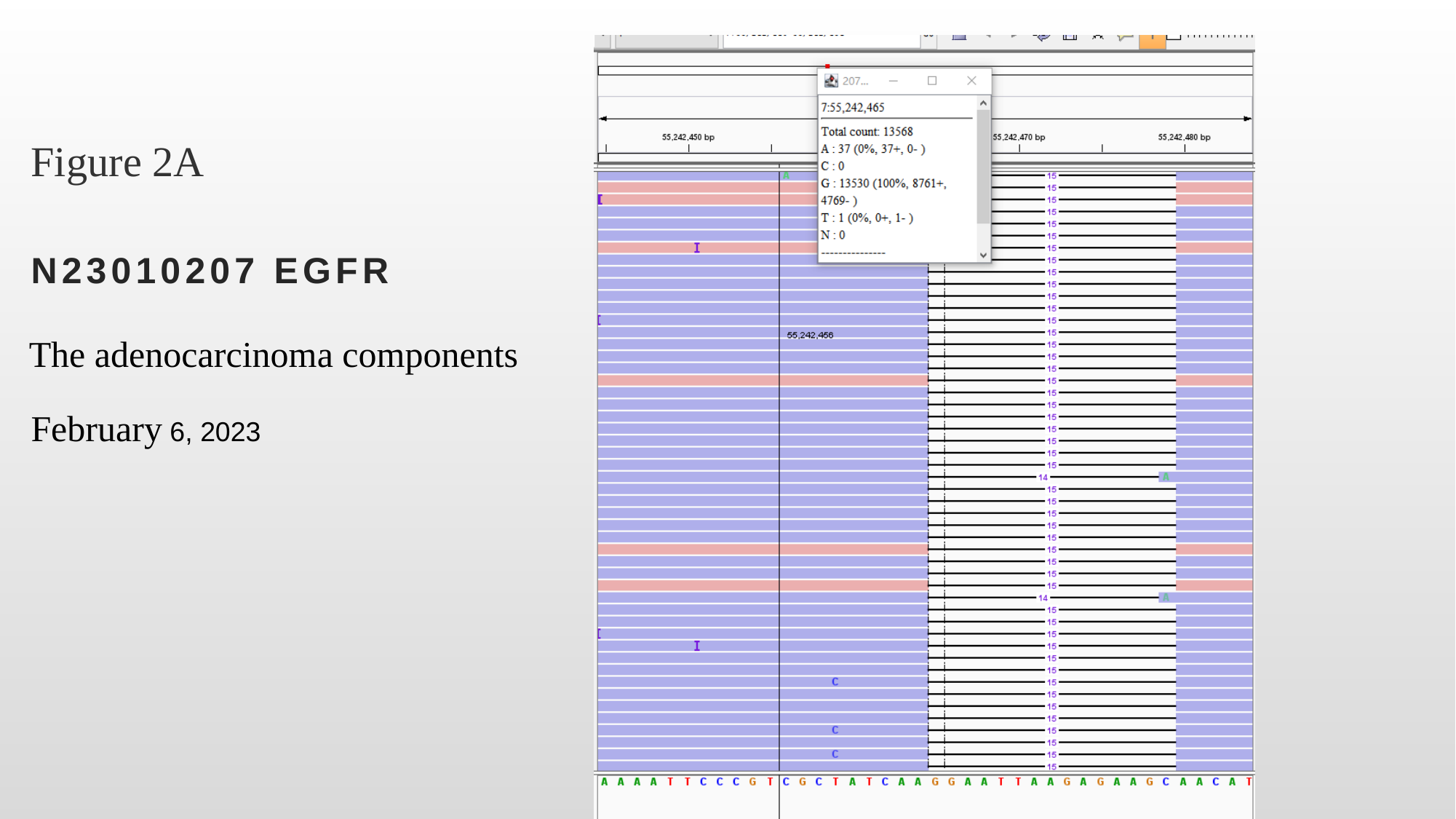

Figure 2A
# N23010207 EGFR
 The adenocarcinoma components
February 6, 2023

## Slide 8
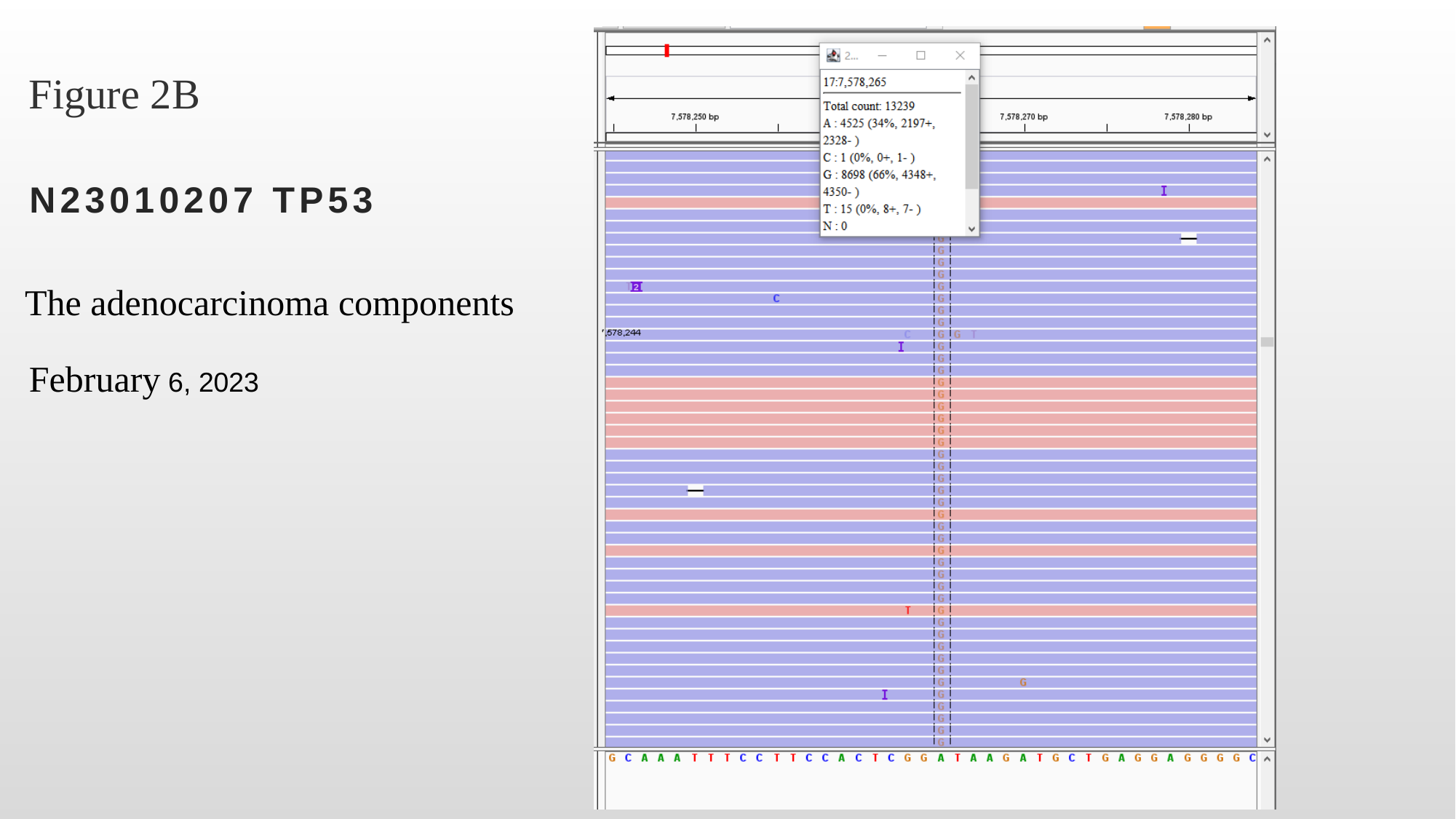

Figure 2B
# N23010207 TP53
 The adenocarcinoma components
February 6, 2023

## Slide 9
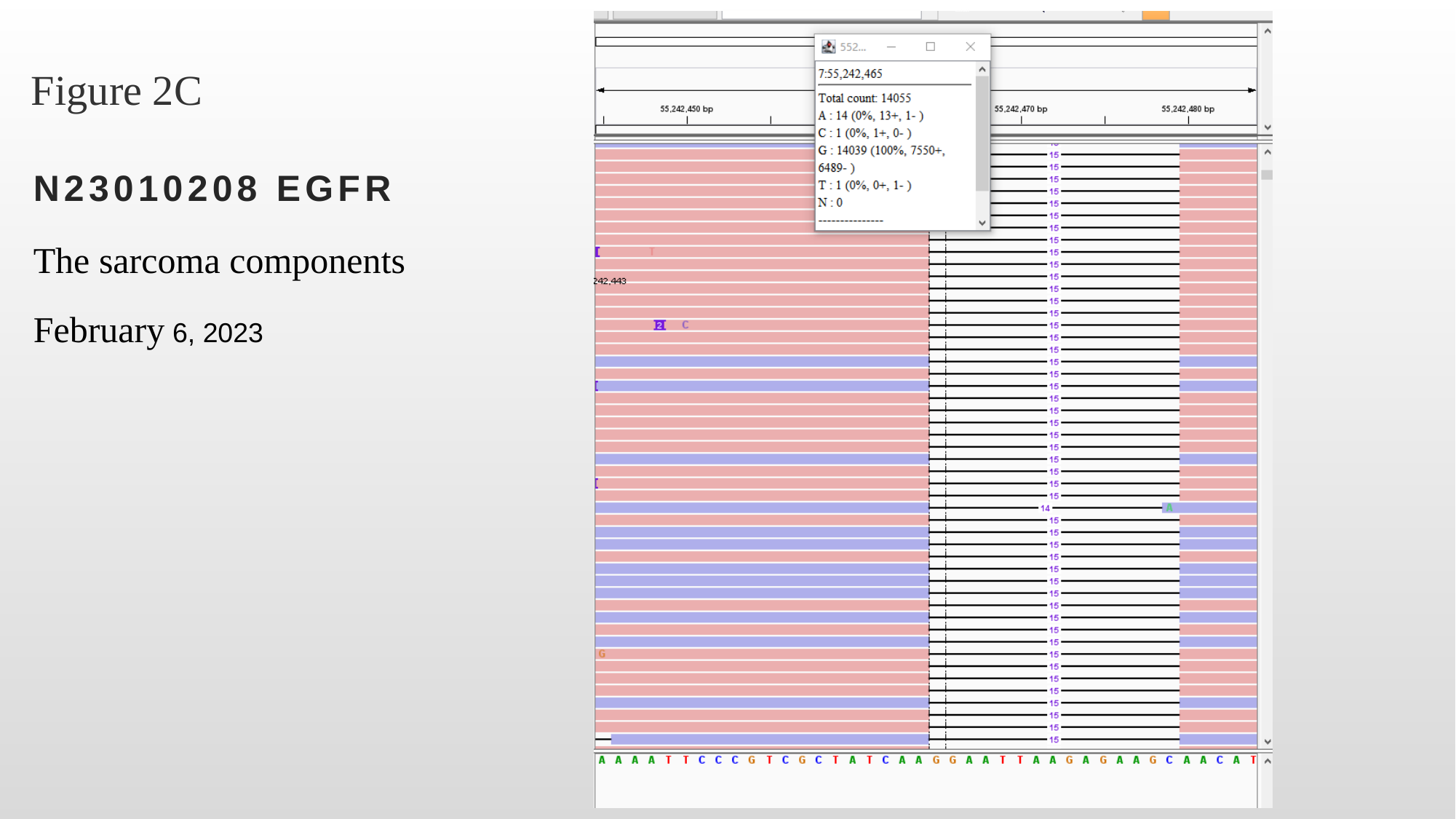

Figure 2C
# N23010208 EGFR
The sarcoma components
February 6, 2023

## Slide 10
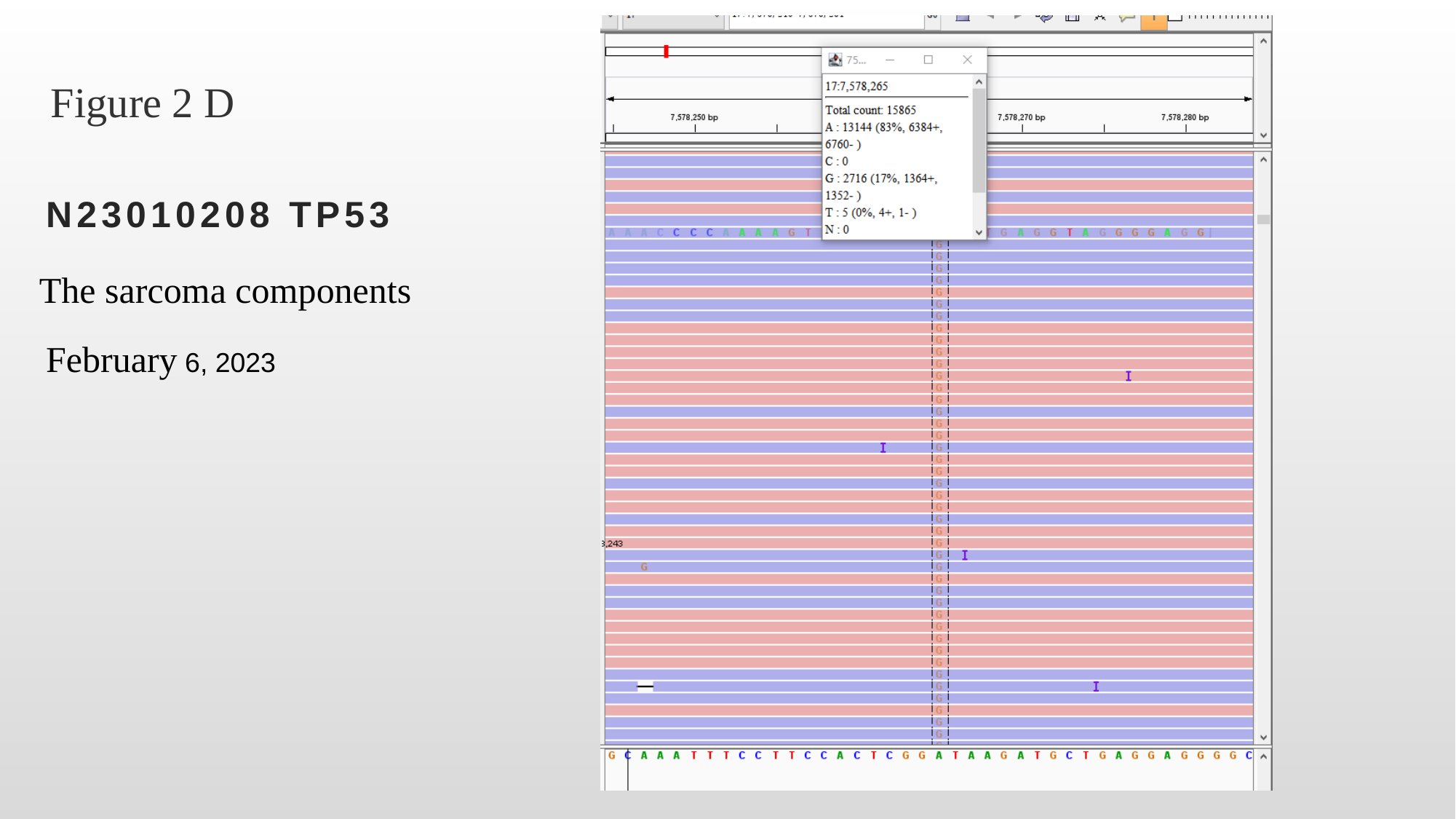

Figure 2 D
# N23010208 TP53
 The sarcoma components
February 6, 2023
